# Alginate hydrogels allow for bioactive and sustained release of VEGF-C and VEGF-D for lymphangiogenic therapeutic applications

**DOI:** 10.1371/journal.pone.0181484

**Published:** 2017-07-19

**Authors:** Kevin T. Campbell, Dustin J. Hadley, David L. Kukis, Eduardo A. Silva

**Affiliations:** 1 Department of Biomedical Engineering, University of California Davis, Davis, California, United States of America; 2 Center for Molecular and Genomic Imaging, University of California Davis, Davis, California, United States of America; Universita degli Studi di Bari Aldo Moro, ITALY

## Abstract

Lymphatic dysfunction is associated with the progression of many cardiovascular disorders due to their role in maintaining tissue fluid homeostasis. Promoting new lymphatic vessels (lymphangiogenesis) is a promising strategy to reverse these cardiovascular disorders via restoring lymphatic function. Vascular endothelial growth factor (VEGF) members VEGF-C and VEGF-D are both potent candidates for stimulating lymphangiogenesis, though maintaining spatial and temporal control of these factors represents a challenge to developing efficient therapeutic lymphangiogenic applications. Injectable alginate hydrogels have been useful for the controlled delivery of many angiogenic factors, including VEGF-A, to stimulate new blood vasculature. However, the utility of these tunable hydrogels for delivering lymphangiogenic factors has never been closely examined. Thus, the objective of this study was to utilize ionically cross-linked alginate hydrogels to deliver VEGF-C and VEGF-D for potential lymphangiogenic applications. We demonstrated that lymphatic endothelial cells (LECs) are sensitive to temporal presentation of VEGF-C and VEGF-D but with different responses between the factors. The greatest LEC mitogenic and sprouting response was observed for constant concentrations of VEGF-C and a high initial concentration that gradually decreased over time for VEGF-D. Additionally, alginate hydrogels provided sustained release of radiolabeled VEGF-C and VEGF-D. Finally, VEGF-C and VEGF-D released from these hydrogels promoted a similar number of LEC sprouts as exogenously added growth factors and new vasculature *in vivo* via a chick chorioallantoic membrane (CAM) assay. Overall, these findings demonstrate that alginate hydrogels can provide sustained and bioactive release of VEGF-C and VEGF-D which could have applications for therapeutic lymphangiogenesis.

## Introduction

Lymphatic vessels are essential for ensuring tissue regeneration and repair due to their critical role in maintaining normal tissue fluid homeostasis. The loss of lymphatic vessel functionality is associated with the progression of several cardiovascular disorders [1–4]. For example, damage to lymphatic vessels in the heart following myocardial infarction can further exacerbate myocardial edema, scarring and ultimately lead to deleterious effects on cardiac function [5–7]. Additionally, lymphatic vessel dysfunction has been associated with the development of atherosclerosis due to their role in mediating reverse cholesterol transport from tissues [2, 3, 8, 9]. Localized stimulation of new lymphatic vessels (lymphangiogenesis) could provide an appealing strategy for reversing the progression of these cardiovascular disorders, which has prompted interest in developing novel strategies for promoting therapeutic lymphangiogenesis.

One promising strategy for promoting therapeutic lymphangiogenesis involves the delivery of pro-lymphangiogenic factors including vascular endothelial growth factor (VEGF) members VEGF-C and VEGF-D. VEGF-C and VEGF-D promote lymphangiogenesis through the binding of vascular endothelial growth factor receptor 3 (VEGFR-3), which is essential for lymphatic vessel development and promotes lymphatic endothelial cell (LEC) proliferation, survival and migration [10–16]. However, members of the VEGF family often experience short half-lives *in vivo* which presents a major challenge for the efficient delivery of VEGF-C and VEGF-D for therapeutic lymphangiogenesis. Many members of the VEGF family, especially VEGF-A, are tightly regulated and have a very short half-live of only a few minutes within tissues limiting the efficacy of exogenous VEGF treatments [17, 18]. Additionally, therapeutics delivered into the body without control of the location or the rate of delivery frequently require large doses to achieve the desired effect which is not only excessive, but can also lead to undesirable or toxic side effects [19].

Polymer based delivery systems can address some of these limitations by providing localized delivery of therapeutics, minimizing required dose, providing temporally controlled release and protecting biological agents from degradation prior to release [19]. In particular, alginate seems to be a promising polymeric biomaterial for delivering VEGF-C and VEGF-D. Alginate is a naturally occurring polysaccharide comprised of both α-L-guluronic (G-block) and β-D-mannuronic (M-block) acid sugar residues. Partial oxidization of alginate can be performed with sodium periodate, which allows the backbone of the polymer chains to become susceptible to hydrolytic degradation [20]. Binary alginate hydrogels are obtained via mixing two molecular weight polymers to achieve varying degradation rates and release properties without sacrificing injectability or mechanical strength [21]. Importantly, these binary alginate hydrogels have been well characterized for the sustained and bioactive delivery of many angiogenic factors, including VEGF-A [22–25]. Altogether, the degradability, injectability and proven utility for controlling VEGF-A delivery make binary alginate hydrogels a promising biomaterial to deliver the related lymphangiogenic VEGF members VEGF-C and VEGF-D.

Therefore, the objective of this study is to assess if alginate hydrogels have utility for delivering VEGF-C and VEGF-D for potential therapeutic lymphangiogenic applications. To our knowledge, the delivery of VEGF-C and VEGF-D from degradable alginate hydrogels has not been evaluated. We hypothesize that LECs will be sensitive to varying temporal presentations of VEGF-C and VEGF-D and that alginate hydrogels will provide sustained and bioactive release of both growth factors. In this work, we begin by observing how LECs respond to varying VEGF-C and VEGF-D temporal profiles through proliferation and 3D sprouting assays. Then, we quantify the release of both VEGF-C and VEGF-D and determine the bioactivity of both growth factors *in vitro* and *in vivo* via both 3D sprouting assays and the chick chorioallantoic membrane (CAM) assay.

## Materials and methods

### Cell culture

Human microvascular lymphatic endothelial cells (LECs) were purchased from commercial sources (Catalog number CC-2810; Lonza). LECs were cultured in EGM-2MV (Lonza) containing EBM-2 with 5% fetal bovine serum (FBS), hydrocortisone, ascorbic acid, human epidermal growth factor (hEGF), GA-1000 antibiotic, VEGF-A, human fibroblast growth factor-beta (hFGF-β) and insulin-like growth factor-1 (IGF-1) as supplied by the vendor’s kit. N media, defined as EGM-2MV without the addition of growth factors, was used as the negative control medium in all experiments.

### Hydrogel formulation

Alginate polymers used in this study were obtained from Novamatrix (FMC). A binary LF 20/40 alginate containing a higher G-block content (> 60% as specified by the manufacturer) was used as the high molecular weight polymer component (HMW; ~250 kDa) and a binary LF10/60 polymer was used as the low molecular weight (LMW; ~50 kDa) for *in vitro* studies. Ultra-pure (UP) alginate polymer was used for the *in vivo* CAM study. UP MVG LMW alginate was prepared as previously described via gamma-irradiating the HMW UP MVG alginate [24, 25]. All alginates had 1% of the sugar residues in the polymer chain oxidized with sodium periodate (Sigma). All oxidized alginate solutions were dialyzed, sterile filtered, lyophilized and stored at -20°C. Hydrogels were prepared by reconstituting alginate in phosphate buffered saline supplemented with calcium and magnesium ions (PBS++; Life Technologies). Hydrogels were prepared to create a final concentration of 2% (w/v) polymer with 75/25 (LMW/HMW) alginate solution and 8.4 mg/mL of calcium sulfate (Sigma). The mixture was then dispensed into rubber molds with approximately 8 mm diameter and 1.5 mm height and incubated for at least 25 minutes at room temperature to ensure full gelation. For hydrogels with FITC-Dextran, 40 μL of FITC-Dextran (3–5 kDa; Sigma) was incorporated into alginate prior to gelation (1 mg/mL). For the *in vitro* studies, lysozyme, VEGF-C and VEGF-D (R&D systems) loaded hydrogels had 5 μL of protein (100 μg/mL in PBS++) added to the alginate prior to gelation (500 ng/mL). For the *in vivo* CAM study, VEGF-A (R&D systems), VEGF-C and VEGF-D had 20 μL of growth factor added to the alginate prior to gelation (2 μg/mL).

### Proliferation assay

LECs (P7) were seeded at 10,000 cells/cm^2^ in 12-well tissue culture plates with serum-free EBM-2 and allowed to adhere for 16 hours. After serum deprivation, conditional medias were added to the cells including EGM-2MV without growth factors supplemented with VEGF-C or VEGF-D at various concentrations (0, 10, 20, 40, 50, 80 or 100 ng/mL). After 4 days with daily media changes, the cells were detached with 0.05% Trypsin-EDTA (Invitrogen). The total number of cells were quantified via a Countess automated cell counter (Life Technologies) and averaged per condition (n = 5–6). The data was then normalized to the no growth factor control (EGM-2MV without the addition of growth factors–N media).

### Sprouting assay

Cytodex 3 microcarrier (MC) beads (GE Healthcare Life Sciences) were prepared and seeded with LECs as previously described in detail [26]. Briefly, MC beads were hydrated overnight at room temperature in DPBS while applying gentle agitation and were then sterilized via autoclaving. Following cooling, the sterile MC beads were mixed with 1.5 mL of LECs (~5 x 10^6^ P7 cells) in EGM-2MV medium. The solution of beads and LECs were transferred to a FACS tube and incubated at 37°C for 4 hours with gentle agitation applied by inverting the tube 3–5 times every 20 minutes. The LEC seeded MC beads were then cultured in T25 flasks on a rotating shaker (~1 rev/sec) at 37°C with daily media changes until approaching 100% confluent. Confluent LEC seeded MC beads were then incorporated within fibrin gels as previously described [24, 27]. Briefly, MC beads were suspended in N media and combined with fibrinogen solution (4 mg/mL in 0.9% NaCl; Sigma) supplemented with aprotinin (~60 μg/mL; Sigma) prior to being aliquoted in 24-well plates. Then a second solution containing thrombin (2.1 U/mL in DPBS; Sigma) was additionally aliquoted at a 4:5 ratio. The plates were then incubated for five minutes at room temperature before being incubated at 37°C for 25 minutes. Gels were then topped with one of the conditional medias daily and incubated for four days. For characterizing the response of LECs to VEGF-C or VEGF-D released from hydrogels, growth factor loaded alginate hydrogels were placed in 24-well plates, topped with N media, incubated at 37°C and then the media was transferred daily to LECs. Equivalent dosages of growth factor were also incubated at 37°C and added to separate LEC groups based on the observed release profiles. After four days of media changes, the gels were washed with DPBS and fixed overnight at 4°C in 4% formaldehyde. The LECs were stained with Hoescht 33342 (Life Technologies) for fluorescent imaging. The total number of confluent beads (n_B_) and sprouts (n_S_) were visually quantified per each well. A sprout was defined as an LEC migrating outwards linearly while remaining anchored to the bead. The average number of sprouts per bead in each gel, n_ave_ = n_S_/n_B_, was calculated and normalized to the average value for the respective negative control wells (n = 3). Representative images of sprout formation were taken at 10X magnification with an inverted fluorescent microscope (Zeiss).

### FITC-Dextran release from hydrogels

Alginate hydrogels were prepared as previous described. FITC-Dextran (3–5 kDa; Sigma) was loaded into hydrogels, the hydrogels (n = 8) were transferred into 24 well plates, topped with 500 μL of PBS++ and incubated at 37°C. At t = 3, 6, 24, 48 and 72 hours the PBS++ was removed from each well and the fluorescent was quantified via a plate reader (Spectramax®i3; Molecular Devices). Each well was topped with fresh PBS++ and returned to incubate at 37°C. Following the collection of data for the last time point, a solution of 0.05 M EDTA in PBS++ is topped in lieu of PBS++ to disintegrate the alginate hydrogels and the remaining FITC-Dextran was quantified via the plate reader to determine the percent of initial released.

### Lysozyme, VEGF-C and VEGF-D release

Iodine-125 (I-125) radiolabeled lysozyme, VEGF-C and VEGF-D were generously provided by the Center for Molecular and Genomic Imaging at UC Davis. I-125 iodinations of VEGF-C and VEGF-D were performed as follows. VEGF-C (50 μg; 3.22 nmol), I-125 (10.3 MBq; PerkinElmer) and carrier potassium iodide (0.15 μg; 6.17 nmol) were combined in PBS (100 μL) in a pre-coated iodination tube (Thermo Scientific Pierce) for 30 minutes at room temperature. The radiochemical yield was >99% by instant thin layer chromatography (ITLC; Biodex). Radiolabeled VEGF-C was purified by centrifuged molecular sieving chromatography (Micro Bio-spin 6; Bio-Rad), with a product yield of 60% (6.2 MBq I-125/30 μg VEGF-C). VEGF-D (50 μg; 3.84 nmol), I-125 (14.1 MBq), and carrier potassium iodide (0.15 μg; 6.17 nmol) were combined in PBS (100 μL) in a pre-coated iodination tube for 30 minutes at room temperature. The radiochemical yield was >99% by ITLC. Radiolabeled VEGF-D was purified by centrifuged molecular sieving chromatography with a product yield of 80% (11.3 MBq I-125/30 μg VEGF-D). Alginate hydrogels were then prepared as previously described with 500 ng/mL of radiolabeled protein. The hydrogels were placed in round bottom tubes (Greiner Bio-One) and topped with 500 μL of PBS++ supplemented with 0.1% bovine serum albumin. At 0.5, 1, 3, 5, 7 and 14 days after starting the release studies, the radiolabeled growth factor present in the buffer solution was removed and measured using a gamma counter (1470 WIZARD; PerkinElmer). All hydrogels were then re-supplemented with a fresh 500 μL of PBS++ with 0.1% BSA and measured with a gamma counter to determine the amount of radiolabeled growth factor retained within the gel (n = 8). A set of standard solutions were also used to calibrate the amount of radiolabeled protein present at each time point.

### Chick chorioallantoic membrane (CAM) assay

The *in vivo* activity of VEGF-C and VEGF-D released from hydrogels were assessed via a modified open-shell CAM assay [28, 29]. Briefly, fertilized hy-line white leghorn chicken eggs (E0) purchased from the UC Davis Avian Facility were incubated horizontally at controlled temperature and humidity (37.8°C with 55–65% humidity) with six rotations per day for 3 days. After 3 days (E3), the eggs were cracked open in a laminar flow hood with aseptic techniques into 88.9 × 88.9 mm weigh boats (Fisher Scientific). Under aseptic conditions, the weigh boats containing the embryos were then incubated at 37.8°C with 60% humidity for an additional week. On day 10 (E10), both blank (negative control) and growth factor loaded hydrogel disks (either VEGF-A, VEGF-C or VEGF-D) were placed on the CAM away from major blood vessels. VEGF-A loaded gels were used as a positive control. Pictures were taken with a 12-megapixel camera (Galaxy S7 edge; Samsung Inc.) around the region of the hydrogel location at 0 and 24 hours of incubation (n = 3). The angiogenic effects were quantified by manually assessing the number of blood vessels surrounding the gel within a 2 mm radius as previously described [30]. The percent change in the number of blood vessels over 24 hours was calculated for each CAM and was normalized to blank controls. Each condition then had the percent difference from the blank control reported. UC Davis Institutional Animal Care and Use Committee (IACUC) Office was formally informed and consulted about the details of the CAM assay used within this work, however CAM assay is UC Davis IACUC exempt. UC Davis is an Office of Laboratory Animal Welfare (OLAW), National Institutes of Health (NIH), Public Health Service (PHS) assured institution (UC Davis Animal Welfare Assurance number is #A3433-01) and follows PHS guidance of the definition for what constitutes a live, vertebrate animal.

### Statistical analysis

Results are shown as the mean values with standard deviations. Experimental conditions were compared to the negative control via a one-tailed unpaired Student’s t-test with an applied Bonferroni correction for the proliferation assays to assess an increase in LEC mitogenesis for any given VEGF-C or VEGF-D dose distribution. A one-way analysis of variance (ANOVA) followed by Tukey’s test was used for the sprouting assays to allow for multiple comparisons between groups. A two-tailed paired Student’s t-test was used for the *in vivo* CAM assay to determine if each growth factor led to an increase in vasculature density. In all cases, significance was asserted at *P* < 0.05. All analyses were performed using GraphPad Prism software (GraphPad Software Inc.).

## Results

### Effect of VEGF-C and VEGF-D dose distribution on LEC proliferation and sprouting

**Fig 1:**
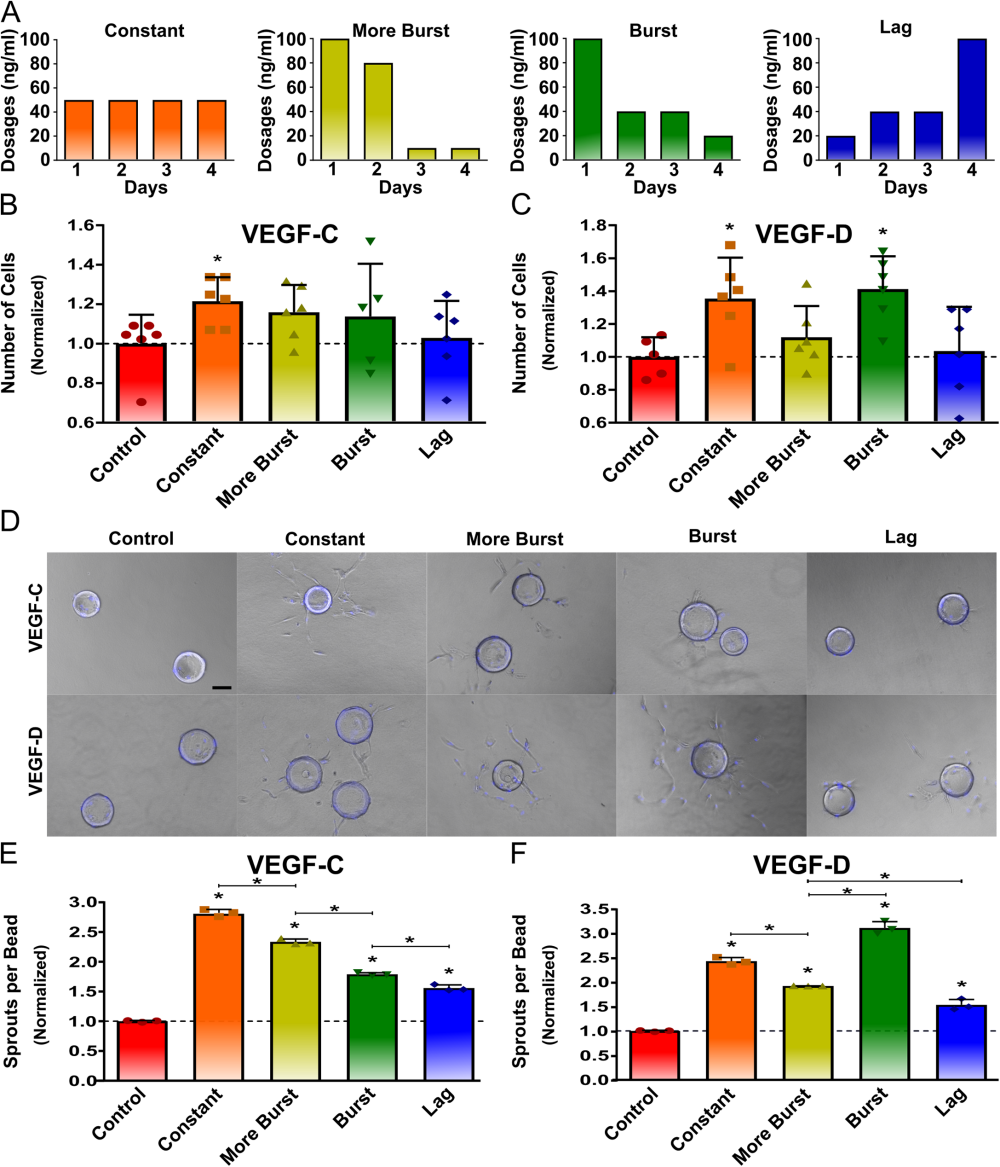
LEC response to VEGF-C and VEGF-D is dependent on dose distributions. LEC proliferation and sprouting response was found to be dependent on the dose distribution of VEGF-C and VEGF-D. The different dose distributions used for VEGF-C and VEGF-D are shown (A). Proliferation was assessed for various dosages of VEGF-C and VEGF-D over four days (B and C). The number of LECs greatly increased in response to a constant dose distribution of VEGF-C and both a burst and constant dose distribution of VEGF-D after four days. Sprout formation was also observed for different dose distributions of VEGF-C and VEGF-D. Scale bar represents 100 **μm** (D). Sprouting assay similarly showed a significant increase in the number of sprouts for all groups, with the most increase for a constant dose distribution of VEGF-C and a burst dose distribution for VEGF-D after four days (E and F). On B, C, E and F, bar represent mean, scatter dot plots display individual measurements and error bars represent standard deviation. (B & C, n = 5–6; E & F, n = 3). Asterisk indicate statistically significant differences (P < 0.05).

To determine how VEGF-C and VEGF-D dose distribution influence LEC response, LECs were cultured with varying dose ranges over four days and were normalized to the no growth factor control. LECs were exposed to the same total VEGF-C or VEGF-D dose (200 ng/mL) over 4 days. Four different dose distributions were used including the constant dose distribution (50 ng/mL every day), the more burst dose distribution (100 ng/mL, 80 ng/mL, 10 ng/mL, 10 ng/mL, from 1–4 days respectively), the burst dose distribution (100 ng/mL, 40 ng/mL, 40 ng/mL, 20 ng/mL from 1–4 days respectively) and the lag dose distribution (20 ng/mL, 40 ng/mL, 40 ng/mL, 100 ng/mL; 1–4 days respectively) (Fig 1A). LECs exposed to a constant VEGF-C dose distribution had approximately 21% more proliferation than the no growth factor control (Fig 1B). LECs exposed to constant and burst VEGF-D dose distributions had approximately 35% and 41% more proliferation than the no growth factor control respectively (Fig 1C). The effects of both VEGF-C and VEGF-D dose distribution on LECs activity/behavior were further assessed using a 3D sprouting assay, which is an *in vitro* model designed to simulate the early events of vessel formation (Fig 1D) [25, 31, 32]. The different VEGF-C and VEGF-D dose distributions tested led to a significant increase in the number of sprouts compared to the no growth factor control. The constant dose distribution led to the most sprouting for VEGF-C, followed by more burst, burst and lag dose distributions (Fig 1E). Additionally, the burst dose distribution led to the most sprouting for VEGF-D, followed by the constant, more burst and lag dose distributions (Fig 1F). Interestingly, LEC sprouting response followed a similar trend to mitogenesis for the tested dose distributions of VEGF-C and VEGF-D.

### Predicting and quantifying I-125 VEGF-C and I-125 VEGF-D release from alginate hydrogels

**Fig 2:**
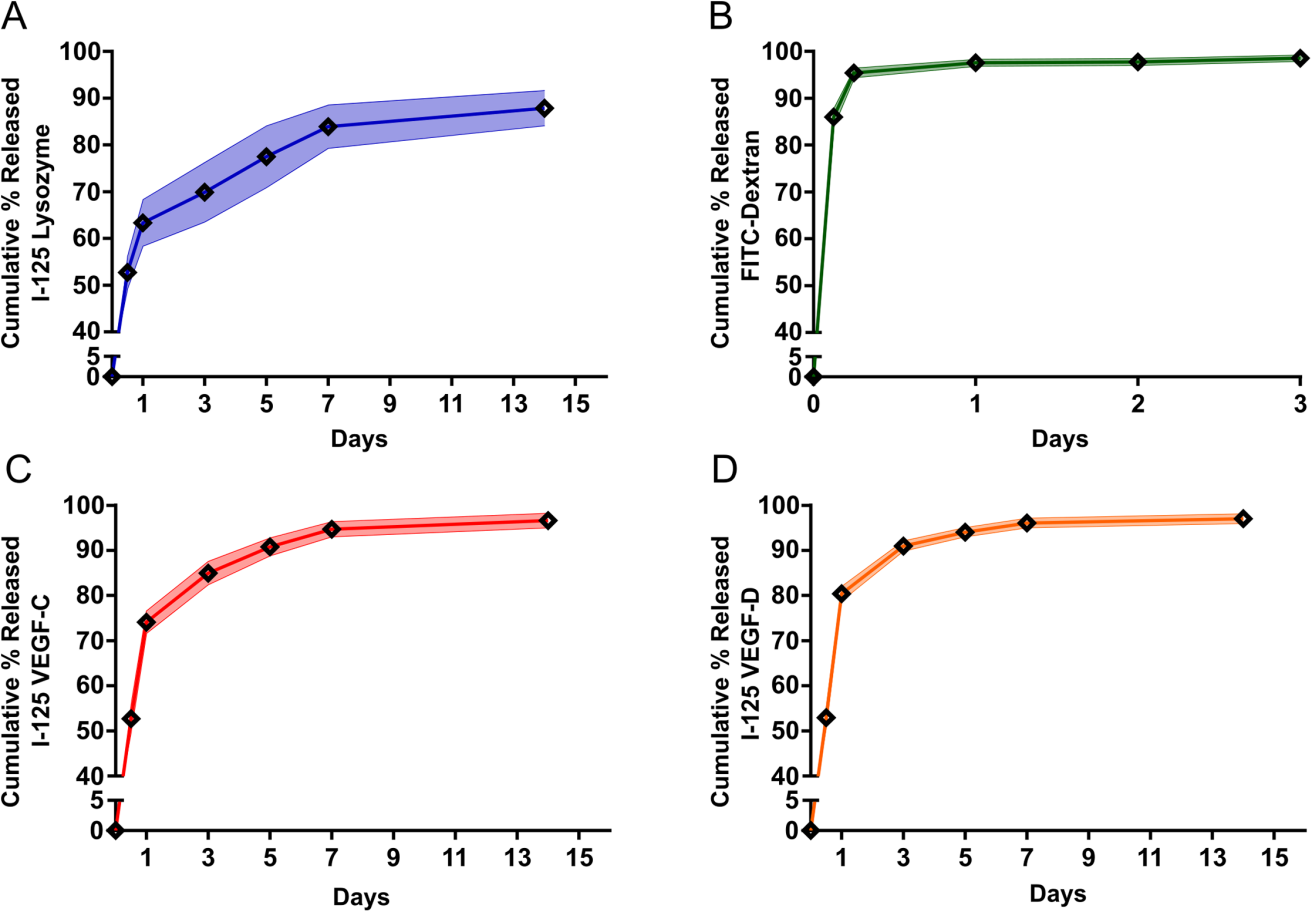
Release profiles of FITC-dextran, I-125 lysozyme, I-125 VEGF-C and I-125 VEGF-D from binary molecular weight alginate hydrogels. I-125 VEGF-C and I-125 VEGF-D release was between the release profiles predicted by comparable size I-125 lysozyme and FITC-dextran. Radiolabeled I-125 Lysozyme of nearly equivalent molecular weight to both growth factors experienced slow release from alginate hydrogels (A). FITC-dextran of comparable hydrodynamic radius to VEGF-C and VEGF-D had very fast release from alginate hydrogels (B). Alginate hydrogels provided sustained release of I-125 VEGF-C and displayed a release profile in between I-125 lysozyme and FITC-dextran (C). I-125 VEGF-D also showed sustained release from alginate with a faster release profile than I-125 VEGF-C (D). Data represent mean ± SD (indicated by shaded areas). (A–D, n = 8).

VEGF-C and VEGF-D were radiolabeled with I-125 in order to determine the *in vitro* release kinetics of these growth factors from alginate hydrogels. An approximation of VEGF-C and VEGF-D release was first obtained using surrogates of comparable hydrodynamic radii prior to quantifying both growth factors release from alginate hydrogels. I-125 radiolabeled lysozyme was used as a surrogate of VEGF-C and had over 90% released within two weeks (Fig 2A). FITC-Dextran was used as a surrogate of VEGF-D and displayed a significantly accelerated release in comparison to lysozyme, with over 90% of the FITC-dextran being released after six hours (Fig 2B). The release of I-125 radiolabeled VEGF-C and VEGF-D from alginate hydrogels was then quantified. Interestingly, VEGF-C released from alginate hydrogels displayed a burst like release profile between FITC-Dextran and lysozyme, leading to over 90% of VEGF-C being released after five days (Fig 2C). In contrast, VEGF-D had faster release than VEGF-C, with over 90% of the initially loaded VEGF-D being released after three days (Fig 2D).

### VEGF-C and VEGF-D released from alginate hydrogels promotes LEC sprouting

**Fig 3:**
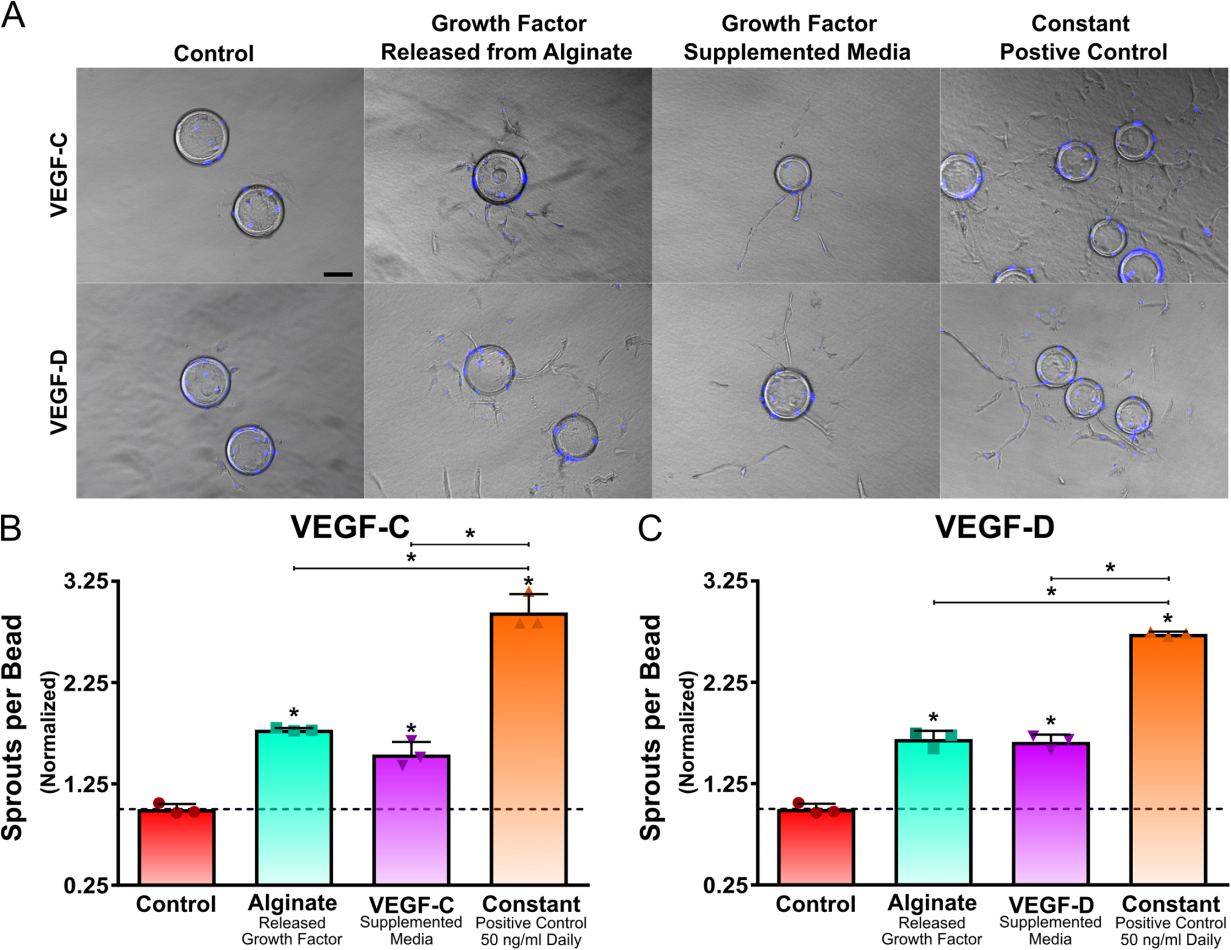
LEC sprouting response to VEGF-C and VEGF-D released from alginate hydrogels. VEGF-C and VEGF-D released from alginate hydrogels promoted LEC sprouting. Representative images of sprouts after four days are shown. Scale bar represents 100 μm (A). Sprouting assay showed that LECs treated with VEGF-C and VEGF-D released from alginate hydrogels led to an increase in sprouts. Additionally, no difference in the number of sprouts between LECs treated with an equivalent exogenous amount of VEGF-C and VEGF-D and LECs treated with VEGF-C and VEGF-D from alginate hydrogels were found (B and C). On B and C bar represent mean, scatter dot plots display individual measurements and error bars represent standard deviation. (B & C, n = 3). Asterisk indicate statistically significant differences (P < 0.05).

The ability of alginate hydrogels to maintain the functionally and bioactivity of released VEGF-C and VEGF-D was assessed via a 3D sprouting assay. LEC groups were exposed to no growth factor (negative control), VEGF-C or VEGF-D released from alginate hydrogels, growth factor supplemented media with equivalent dosages predicted from the radiolabeled release profile, or a constant dose (positive control, 50 ng/mL of VEGF-C or VEGF-D every day) for four days (Fig 3A). VEGF-C and VEGF-D released from alginate hydrogels over the course of four days were found to stimulate LEC sprouts. Alginate released VEGF-C promoted a slightly increased, though not significant, number of sprouts than VEGF-C supplemented media with approximately a 1.8 and 1.5-fold increase in the number of sprouts compared to the no growth factor condition respectively (Fig 3B). Both alginate released and VEGF-D supplemented media promoted a similar number of sprouts, with approximately 1.7 and 1.6-fold increase in the number of sprouts compared to the no growth factor condition respectively (Fig 3C). As expected, the constant dose distribution led to the most sprouting for both VEGF-C and VEGF-D.

### Alginate hydrogels loaded with VEGF-C and VEGF-D promoted new vasculature in a CAM assay

**Fig 4:**
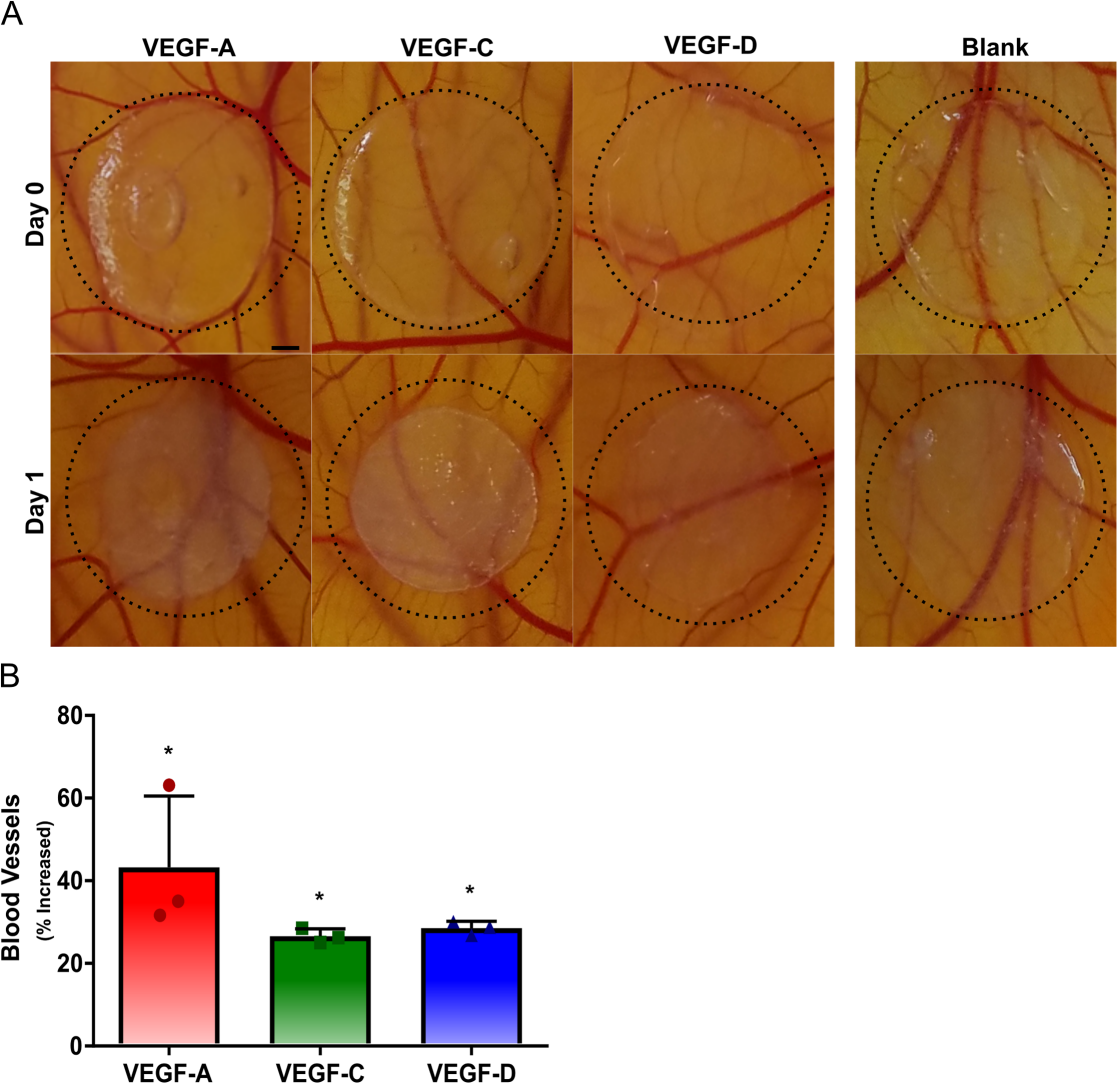
VEGF-C and VEGF-D release from alginate hydrogels stimulates new vasculature in an *Ex Ovo* CAM assay. Sustained delivery of VEGF-A, VEGF-C and VEGF-D induced blood vessel formation in a CAM assay. Representative images of the hydrogels placed on the CAMs at 0 h and after 1 day of incubation are shown. Scale bar represents 1 mm (A). Quantified blood vessel development showed that all growth factors resulted in a statistically significant increase in vasculature density (B). On B bar represent mean, scatter dot plots display individual measurements and error bars represent standard deviation. (B, n = 3). Asterisk indicate statistically significant differences (P < 0.05).

Additional bioactivity of VEGF-C and VEGF-D released from alginate hydrogels were assessed *in vivo* using a CAM assay. Alginate hydrogels loaded with VEGF-A (positive control), VEGF-C and VEGF-D were placed on separate CAMs with a blank hydrogel (Fig 4A). VEGF-A, VEGF-C and VEGF-D led to approximately 43%, 26% and 28% increase in new vasculature within the CAMs when compared to the blank control (Fig 4B). Furthermore, no significant difference in the number of new blood vessels were observed for any of the three growth factors.

## Discussion

This study investigates the utility of an injectable and degradable binary alginate hydrogel system for delivery of the pro-lymphangiogenic factors VEGF-C and VEGF-D. This work demonstrates that human LECs are sensitive to temporal profiles of VEGF-C and VEGF-D and that alginate hydrogels can provide a sustained presentation of these factors. Moreover, this work also shows that VEGF-C and VEGF-D released from alginate hydrogels stimulate both 3D LEC sprouting *in vitro* and new vasculature *in vivo* via a CAM assay. Overall, this is the first study characterizing VEGF-C and VEGF-D release from injectable and degradable binary alginate hydrogels for potential lymphangiogenic therapeutic applications.

The results of this study confirmed that *in vitro* LEC response is dependent on VEGF-C and VEGF-D dose and temporal presentation. Constant dosages of VEGF-C and VEGF-D have been shown to promote LEC proliferation and survival [15, 33], though the importance of their temporal gradients has thus far been neglected. This study showed that a constant dose distribution was the only dose profile that led to an increase in LEC proliferation for VEGF-C, while both a burst or constant dose distribution increased LEC proliferation for VEGF-D. The effect of VEGF-C and VEGF-D dose distributions on LECs sprouting were also tested via a 3D sprouting assay. The 3D sprouting assay is an *in vitro* model designed to allow dynamic vessel formation throughout an embedded gel to simulate the early events of blood vessel formation [25, 31, 32]. Recent work has also shown applications of these assays for simulating the initial stages of lymphangiogenesis [34, 35]. We showed that the VEGF-C and VEGF-D dose distributions led to sprouting, which agrees with previous studies showing that VEGF-C promotes LEC sprout invasion *in vitro* [35, 36]. Furthermore, a constant concentration of VEGF-C and high levels of VEGF-D at early time points followed by a gradually decreasing concentration led to the most LEC sprouting. Interestingly, LEC sprouting followed the same trend as proliferation for the different dose distributions of VEGF-C and VEGF-D. Taken together, these results show that controlling the temporal presentation of VEGF-C and VEGF-D is important for stimulating the lymphangiogenic potential of LECs and further highlights applications of 3D sprouting assays for studying the initial formation of lymphatic vessels.

Multiple factors affect the release kinetics of therapeutic cargos from hydrogels including therapeutic and polymer interactions and hydrogel degradation [19, 37]. Given that VEGF-C and VEGF-D are predicted to be significantly smaller than the typical pore size of alginate hydrogels [38, 39], it has been suggested that the release kinetics will be primarily mediated via interactions between the factors and alginate polymer [19, 37]. Therefore, lysozyme and FITC-Dextran with similar hydrodynamic radii to VEGF-C and VEGF-D respectively were used as surrogates to predict the release of both growth factors. The hydrodynamic radii of VEGF-C, VEGF-D and lysozyme were obtained using the vendor specified molecular weight of the globular proteins as previously described [39]. Interestingly, VEGF-C experienced significantly accelerated release than lysozyme despite both proteins having a predicted hydrodynamic radius of 2.0 nm. This is possibly due to lysozyme having a higher isoelectric point than VEGF-C and therefore increased affinity to the anionic alginate polymer. Furthermore, VEGF-D experienced significantly delayed release compared to FITC-Dextran. This could be due to VEGF-D’s larger predicted hydrodynamic radius of 1.8 nm compared to FITC-Dextran’s vendor specified hydrodynamic radius of 1.4 nm. Interestingly, this study highlights potential steric and affinity properties to consider for predicting VEGF-C and VEGF-D release from alginate hydrogels.

Injectable and degradable binary alginate hydrogels have been validated for the delivery of many angiogenic factors, including VEGF-A [22–25], IGF-1 [40], platelet derived growth factor-BB (PDGF-BB) [23], sphingosine-1-phosphate (S1P) [26, 29] and stromal cell derived factor-1 (SDF-1) [22], though the release of lymphangiogenic factors have not been widely studied. Previous work has shown that various forms of VEGF-C delivered from gelatin [41, 42] and albumin alginate [6] promoted lymphangiogenesis *in vivo*. However, the use of injectable and degradable binary alginate hydrogels to deliver bioactive and temporally controlled VEGF-C and VEGF-D has not been specifically tested yet. These alginate hydrogels were found to provide continuous release of both VEGF-C and VEGF-D over a week. Interestingly, VEGF-C experienced significantly delayed release compared to VEGF-D. VEGF-C is predicted to have a higher isoelectric point and hydrodynamic radius than VEGF-D and agrees with our previous observations. Thus, injectable and degradable binary alginate hydrogels provided a sustained release of VEGF-C and VEGF-D.

VEGF-C and VEGF-D released from injectable and degradable binary alginate hydrogels also maintain their biological activity by simulating LEC sprouts *in vitro* and new vasculature *in vivo* via a CAM assay. Interestingly, VEGF-C and VEGF-D released from alginate hydrogels over the course of four days promoted LEC sprouting and led to a similar number of sprouts as LECs treated with an equivalent dose of growth factor based on their release profiles. The bioactivity of VEGF-C and VEGF-D released from alginate hydrogels were further assessed by their ability to stimulate new blood vasculature *in vivo* via an *ex ovo* CAM assay. Fully mature VEGF-C and VEGF-D activate vascular endothelial growth factor receptor-2 (VEGFR-2) to promote new blood vessels [10, 43–45] and VEGF-C has been shown to promote angiogenesis in CAM assays [46]. VEGF-A was used as the positive control because it is a known potent angiogenic promoter in CAM assays [47–50]. VEGF-A, VEGF-C and VEGF-D were all found to lead to a significant increase in blood vessels. Taken together, degradable alginate hydrogels can release bioactive VEGF-C and VEGF-D and thus have promising applications for therapeutic lymphangiogenesis.

In summary, this work shows that degradable alginate hydrogels allow for sustained and bioactive release of the lymphangiogenic factors VEGF-C and VEGF-D. Specifically, this study shows injectable and degradable alginate hydrogels as a promising biomaterial for delivering active VEGF-C and VEGF-D, which could have future therapeutic lymphangiogenic applications.
